# Epigenetic Requirements for Triggering Heterochromatinization and Piwi-Interacting RNA Production from Transgenes in the *Drosophila* Germline

**DOI:** 10.3390/cells9040922

**Published:** 2020-04-10

**Authors:** Pavel A. Komarov, Olesya Sokolova, Natalia Akulenko, Emilie Brasset, Silke Jensen, Alla Kalmykova

**Affiliations:** 1 Institute of Molecular Genetics, Russian Academy of Sciences, Moscow 123182, Russia; 2 Present address: Friedrich Miescher Institute for Biomedical Research, Maulbeerstrasse 66, 4058 Basel, Switzerland; 3 GReD, Université Clermont Auvergne, CNRS, INSERM, Faculté de Médecine, TSA 50400, 63001 Clermont-Ferrand, France

**Keywords:** retrotransposon, transgene, piRNA cluster, HP1, Rhino, H3K9me3, maternal inheritance, convergent transcription, *Drosophila*

## Abstract

Transgenes containing a fragment of the *I* retrotransposon represent a powerful model of piRNA cluster *de novo* formation in the *Drosophila* germline. We revealed that the same transgenes located at different genomic loci form piRNA clusters with various capacity of small RNA production. Transgenic piRNA clusters are not established in piRNA pathway mutants. However, in the wild-type context, the endogenous ancestral *I*-related piRNAs heterochromatinize and convert the *I*-containing transgenes into piRNA-producing loci. Here, we address how the quantitative level of piRNAs influences the heterochromatinization and piRNA production. We show that a minimal amount of maternal piRNAs from ancestral *I-*elements is sufficient to form the transgenic piRNA clusters. Supplemental piRNAs stemming from active *I*-element copies do not stimulate additional chromatin changes or piRNA production from transgenes. Therefore, chromatin changes and piRNA production are initiated by a minimum threshold level of complementary piRNAs, suggesting a selective advantage of prompt cell response to the lowest level of piRNAs. It is noteworthy that the weak piRNA clusters do not transform into strong ones after being targeted by abundant *I*-specific piRNAs, indicating the importance of the genomic context for piRNA cluster establishment. Analysis of ovarian transcription profiles suggests that regions facilitating convergent transcription favor the formation of transgenic piRNA clusters.

## 1. Introduction

Piwi interacting RNAs (piRNAs) play a key role in silencing of Transposable Elements (TEs) to prevent their excessive transposition in the germline. piRNAs have endogenous origin and are generated from long transcripts derived from distinct genomic regions termed piRNA clusters [1,2]. Specific chromatin components of piRNA clusters along with RNA processing/export factors ensure the particular fate for piRNA precursors. In *Drosophila*, Rhino (Rhi) protein, a germline-specific ortholog of Heterochromatin Protein 1 (HP1), is considered as a key factor of piRNA cluster chromatin. Binding of Rhi mediates non-canonical transcription and processing of the piRNA precursors emerging from piRNA clusters [3,4,5]. In germ cells, in contrast to protein-coding mRNAs, piRNA precursors are delivered to the piRNA processing machinery localized in the specialized cytoplasmic structure, “nuage”, and on the outer mitochondrial membrane [6,7,8,9]. piRNA-mediated silencing is a potent mechanism of expression control realized through transcriptional [10,11] and co-transcriptional [12] repression of target loci. Despite the high regulatory potential of piRNAs, their practical application in the suppressing of the target gene expression is complicated by the fact that little is known about the prerequisites for the formation of a robust piRNA cluster.

Endogenous piRNA clusters have species-specific structural organization; however, all of them are the sources of small RNAs interacting with PIWI subfamily Argonaute proteins [13,14]. In *Drosophila*, there are several types of germline piRNA clusters. Heterochromatic regions enriched by TE fragments represent pericentromeric piRNA clusters [1]. Non-canonical widespread transcription initiation is a peculiar feature of pericentromeric piRNA clusters [3]. *Drosophila* telomeric regions, as well as euchromatic copies of TEs also produce piRNA precursors [15,16]; however, their transcription is driven by canonical promotors of active TEs and telomeric repeats. This type of piRNA cluster is similar to mammalian pachytene piRNA clusters and individual retrotransposon copies generating primary piRNAs [2,17]. Therefore, the essential question is what is the role of epigenetic factors to form an active piRNA cluster. The study of endogenous piRNA clusters is seriously complicated by their enrichment by repeats and the high level of intra-species polymorphism. The discovery of transgene-associated dual-strand piRNA clusters [18,19] in *Drosophila* significantly promoted piRNA cluster research in the *Drosophila* germline.

Significant insight into the piRNA cluster formation came from the discovery of paramutation phenomenon related to the formation of transgenic piRNA clusters. A transgenic locus, initially non-piRNA-producing, targeted by complementary piRNAs derived from a homologous transgene undergoes paramutagenic transformation accompanied by chromatin reorganization, resulting in stable production of small RNAs [18]. In this case, piRNAs govern the conversion of a homologous genomic locus into a piRNA-producing region followed by transmission of this state over generations. Maternal inheritance of piRNAs maintains the piRNA-producing capacity of the paramutagenized locus.

**Figure 1:**
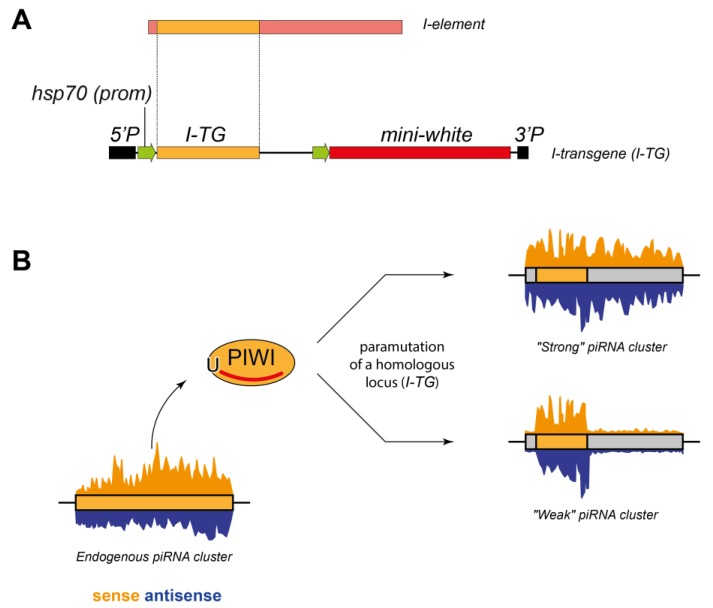
An experimental system of the transgenes containing the *I*-element fragment. (**A**) Scheme of the *I-TG* transgene containing 5′ and 3′ P-element arms, two *hsp70* promoters (green arrows), *mini-white*, and *I*-element fragment. (**B**) Two scenarios of the paramutation of the *I-TG* transgenes leading or not to the formation of a piRNA cluster in the germline of the *Drosophila* ovary. To mark the difference between the epigenetically distinct parts of the transgenes, the fragment of an endogenous piRNA cluster (i.e., *I-*element fragment) is colored yellow, whereas the rest of the transgene in grey.

Previously, we developed a powerful model of germline piRNA clusters [19] based on the *I-TG* transgenes containing a fragment of the *I*-element, a *Drosophila* LINE (Long Interspersed Nuclear Element) transposon (Figure 1A) [20,21]. From the perspective of the presence of I-elements, two types of Drosophila melanogaster strains exist: Inducer (I) strains, which possess functional *I*-elements, and Reactive (R) strains devoid of such elements. Both I and R strains contain non-functional *I*-elements within heterochromatic regions from an ancestral *I*-element invasion. Originally, the R strain *w^K^* devoid of active *I*-element copies was used for transgenesis. We revealed that the same transgenes, located at different euchromatic genomic loci, form piRNA clusters with various abilities of small RNA production, termed as “weak” and “strong” transgenic piRNA clusters (Figure 1B) [19,22]. Most importantly, “strong” piRNA clusters are highly enriched in Rhi and produce piRNAs from all transgenic parts, in contrast to “weak” ones, whose piRNAs mostly map to the *I*-element fragment.

Presumably, piRNAs originating from the ancestral *I*-elements and complementary to the transgenic *I*-element fragment drive this transformation. However, only “strong” transgenic piRNA clusters generate small RNAs from all parts of the transgene and bind Rhi. Other *I-TG* transgenes referred to as “weak” are enriched with HP1 and H3K9me3, but not Rhi, and do not produce a substantial amount of piRNAs, apart from the *I*-element fragment. In this case, endogenous *I*-specific piRNAs are sufficient to start the ping-pong amplification cycle [1,23] by cleaving transgene transcripts. Resulting *I*-specific piRNAs are responsible for the suppression of *I*-element activity [19,20]. Since the *I-TG* transgenes demonstrating different scenarios were inserted into various euchromatic regions, the importance of the genomic context for the transgenic piRNA cluster establishment was proposed [22].

The *I-TG* transgenic model provided a unique opportunity for investigating the transcriptional changes accompanying *de novo* piRNA cluster formation. *I-TG*s are related to dual-strand clusters, which are transcribed and produce piRNAs from both DNA strands. We have shown that the establishment of a transgenic dual-strand piRNA cluster leads to the activation of transcription from both genomic strands initiated at multiple random sites [22]. Notably, launching of even a low level of antisense transcription provides the generation of piRNA precursors and the formation of “strong” dual-strand piRNA clusters.

In this study, we aim to determine the quantitative threshold of maternal piRNAs required for the epigenetic changes of the complementary locus in the progeny. Do the chromatin structure and small RNA production from the transgenes depend on the amount of endogenous *I*-specific piRNAs targeting the transgenes? We found that the increase in the level of piRNAs targeting *I-TG* transgenes does not stimulate additional changes of the chromatin state or piRNA production by the transgenes. The study of paternal transgene inheritance by crossing transgenic males with *w^K^* females whose oocyte cytoplasm is the poorest in *I*-specific piRNAs [24] suggests that heterochromatinization and piRNA production at the target locus are activated by a minimum threshold level of complementary piRNAs.

## 2. Materials and Methods

### 2.1. Drosophila Melanogaster Transgenic Strains

Transgenic strains here referred to as 1.9R, 2.1R, 2.4R, and 3.6R bearing the *I*-element fragment have previously been described [19,20,22]. In brief, a fragment of the *I-*element corresponding to the 167-2484 nucleotides of the M14954 GenBank sequence was cloned into the pW8-hsp-pA vector containing two *hsp70* promoters, which drive the expression of the (1) *I-*element fragment and (2) *mini-white* gene. The *I*-element fragment was cloned in sense (1.9, 2.1, 2.4) or antisense (3.6) orientations relative to the *hsp70* promoter. The transgene referred to as *I-TG* was inserted randomly into the genome of the *w^K^* R strain via a *P*-element-mediated transformation (Figure 1A). Genomic locations of the transgenes in the independent transgenic strains are provided in Table S1. *I-TG* transgenes referred to as “weak” or “strong” are designated by a superscript ^w^ or ^s^, respectively.

To create transgenic strains with the I-background, we crossed *w^K^*-derived males carrying the transgenes with *w^1118^; CyRoi/+;TM3, Sb Ser/+* females, which contain functional *I*-elements. Then, the F1 progeny females and F1 males carrying the balancer chromosomes and the *I-TG*s were crossed. Among the F2 progeny, we selected the transgenic flies not carrying the balancer chromosomes and subsequently established “I” transgenic lines (1.9 ^w^I, 2.1 ^s^I, 2.4 ^s^I, 3.6 ^w^I), which now should have the transgene together with functional *I*-elements. To check the effective presence of functional *I*-elements, a test of the inducer phenotype for these transgenic “I” strains was performed by crossing transgenic “I” males with *w^K^* females and measuring the percentage of dead embryos laid by the progeny according to a previously described procedure [21]. In this test, a high percentage of non-hatched embryos from this progeny indicated the presence of functional *I*-elements in the crossed fathers.

1.9^w^mat, 2.1^s^mat, and 2.4^s^mat correspond to the progeny obtained by crossing 1.9^w^R, 2,1^s^R, 2.4^s^R females with *w^K^* males (“maternal” transgene transmission) and 1.9^w^pat, 2.1^s^pat, and 2.4^s^pat correspond to the progeny obtained by crossing *w^K^* females with 1.9^w^R, 2.1^s^R, 2.4^s^R males (“paternal” transgene transmission).

For the study in mutant background for *spindle-E,* we used *spnE^1^* and *spnE^hls3987^* mutant alleles.

### 2.2. Small RNA Library Preparation and Analysis

Small RNAs, 19-29 nt in size, from total ovarian RNA extracts were cloned as previously described in [19] and sequenced using the Illumina HiSeq 2500 and NovaSeq 6000 systems. After clipping the Illumina 3′-adapter sequence with Cutadapt [25], small RNA reads that passed quality control and the minimal length filter (>18nt) were mapped (allowing 0 mismatches) to the *Drosophila melanogaster* genome (April 2006, BDGP assembly R5/dm3) or transgenes by bowtie2 [26]. Small RNA counts were normalized to the library depth. The ping-pong signature was analyzed as described in [27]. Size distributions and read coverage of small RNAs were calculated using SAMtools [28] and BEDtools [29] and visualized using custom R scripts [30] and the ggplot2 package [31]. Determination of the counts of TE-specific reads was performed by sRNAPipe [32] using a canonical set of *Drosophila melanogaster* TEs from RepBase [33].

Ovarian small RNA-seq data for *w^K^*, *w^1118^; CyRoi/+;TM3, Sb Ser/+,* 2.4;*spnE/*+, 2.4;*spnE/spnE*, 1.9I, 1.9R, 2.1I, 2.1R, 2.4I, 2.4R, 3.6I, 3.6R, 1.9mat, 1.9pat, 2.1mat, 2.1pat, 2.4mat, and 2.4pat *Drosophila* lines are available at Gene Expression Omnibus (GEO), Accession Number GSE138886.

### 2.3. GRO-seq Library Preparation and Analysis

Run-on transcription and preparation of ovarian Global Run-On sequencing (GRO-seq) libraries was performed as previously described [10,34]. Three-hundred pairs of ovaries were dissected from F1 females after paternal/maternal inheritance of the 1.9 *I-TG* transgene. GRO-seq reads were mapped to the R5/dm3 genomic assembly or transgene sequence by bowtie2 and de-duplexed. rRNA reads were excluded from the GRO-seq data before analysis. GRO-seq data for 1.9mat and 1.9pat were deposited in Gene Expression Omnibus (GEO), Accession Number GSE138886.

### 2.4. Chromatin Immunoprecipitation

ChIP was performed according to a published procedure [22] with modification. After deproteinization, DNA was cleaned using a CleanMag DNA (Evrogen, Russia). Chromatin was immunoprecipitated with the following antibodies: anti-HP1a (C1A9 Developmental Studies Hybridoma Bank), anti-histone H3 (tri methyl K9) (Millipore 07-523), and Rhi antiserum [35]. The primers used in this study are listed in Table S5. Quantitative PCR (qPCR) was conducted with a Light cycler 96 (Roche). The obtained values were normalized to the input and compared with the values for the *rp49* gene as a control genomic region. The standard error of the mean (SEM) of triplicate PCR measurements for three biological replicates was calculated.

## 3. Results

### 3.1. piRNAs Are Required for the piRNA Production and Maintenance of the Heterochromatic State at Transgenic piRNA Clusters

**Figure 2:**
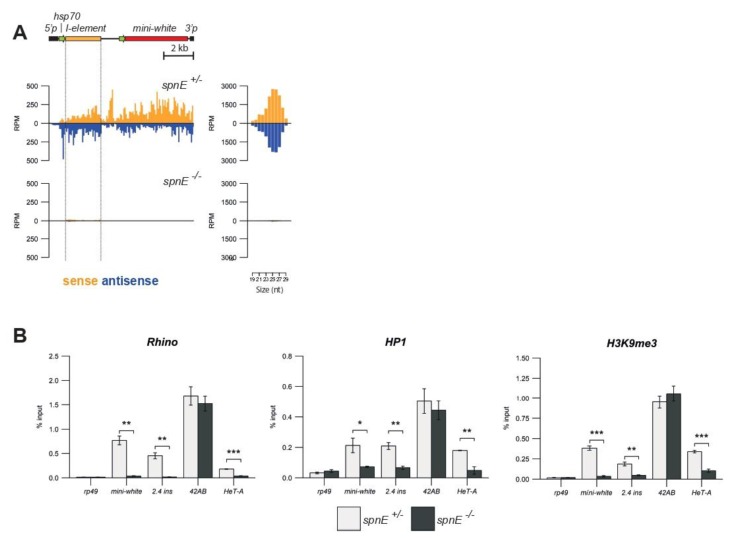
piRNA pathway disruption caused the loss of the heterochromatic state and piRNA production at transgenic piRNA clusters. (**A**) Normalized numbers of ovarian small RNAs (19-29 nt) mapped to transgenic constructs in hetero- and transheterozygous *spnE* mutants (brown—sense; blue—antisense; no mismatches allowed). The scheme of the *I-TG* transgene is shown above. The length distribution of transgenic small RNAs is shown to the right. (**B**) HP1, H3K9me3, and Rhi occupancy was estimated by ChIP-qPCR using primers corresponding to the indicated unique transgenic regions (* *p* < 0.05 to 0.01, ** *p* < 0.01 to 0.001, *** *p* < 0.001, *t*-test). Chromatin analysis of a unique region of the *42AB* pericentromeric piRNA cluster and telomeric *HeT-A* retroelements was performed for comparison. The *rp49* gene was used as a negative control. RPM, Reads Per Million.

Chromatin of all germline piRNA clusters was enriched in H3K9me3 histone markers and Rhi and HP1 proteins, although the mechanisms of chromatin state maintenance were not always the same for different clusters. piRNAs are required for the establishment, but not for maintenance, of compact chromatin at pericentromeric piRNA clusters [36]. In contrast, piRNAs are required for the maintenance of the heterochromatin markers at telomeric piRNA clusters [15]. In order to investigate the role of the piRNA pathway in the formation of transgenic piRNA clusters, we analyzed small RNA production and the chromatin state of *I*-transgenes in the background of a *spindle-E* mutant *(spnE)*. The *spnE* gene encodes the RNA helicase SpnE, one of the key piRNA processing factors [37]. We generated a strain bearing the “strong” *I-TG* transgene from the 2.4^s^ strain and *spnE* mutations. Small RNA sequencing revealed that *spnE* mutations resulted in a substantial reduction of transgene-derived small RNAs (Figure 2A), which confirmed that the *I-TG* in the 2.4^s^ strain was indeed a genuine piRNA cluster. To examine how *spnE* mutations affect the enrichment of H3K9me3 and the deposition of HP1 and Rhi at the *I-TG*, we performed ChIP-qPCR. Primers specific to the region upstream of the transgene insertion site or to the transgenic *mini-white*, but not to the P-element arms because of the presence of the P-element-based construct in the *spnE* mutant locus (*spnE^hls3987^* allele), were used. We observed a dramatic decrease in the enrichment of HP1, H3K9me3, and Rhi in the transgenic regions in the ovaries of the *spnE* mutants. Loss of HP1 and Rhi was also demonstrated for the *HeT-A* telomeric retrotransposons, as expected [15]. However, no significant changes were observed for the pericentromeric cluster *42AB* (Figure 2B). According to the previously published data, piRNAs are required at early embryogenesis for the loading of Rhi and H3K9me3 at the pericentromeric dual-strand piRNA clusters, but in the ovaries of adult flies, the chromatin of such piRNA clusters is maintained by an unknown Piwi-independent mechanism [36]. These results argue that *I-TGs* are different from pericentromeric dual-strand piRNA clusters and belong to a separate group of piRNA clusters, which require endogenous piRNAs for the maintenance of their chromatin during oogenesis.

### 3.2. Small RNA Production and Chromatin Structure of the I-TG Transgenes Are Not Changed upon the Increase of I-Specific piRNAs Targeting the Transgenes

The current model for the establishment of transgenic piRNA clusters implies the interaction of endogenous piRNAs with the complementary transcripts of the target locus (reviewed in [38]). The key question is the amount of piRNAs required to trigger transcriptional silencing and/or piRNA production. The *Drosophila I-TG* transgenic model provides an opportunity to address this question. *I-TG* transgenes were originally established in the R background of the *w^K^* strain lacking functional *I*-elements, but containing fragments of ancestral *I*-related copies [20,21]. In ovaries of the *w^K^* strain, the level of *I*-specific piRNAs that could target the *I-TGs* without mismatch was extremely low and estimated as 28 RPM (Reads Per Million) (Table S3). In addition, the *I-TG* construct contains two fragments of *hsp70* promoters. Endogenous *hsp70* gene loci represent piRNA clusters producing abundant piRNAs [19]. Moreover, a recent study provided evidence for *hsp70* piRNA-guided transcriptional silencing of the UASt promoter in the *Drosophila* germline [39].

The level of *hsp70*-specific piRNAs that could target the *I-TGs* in the ovaries of the *w^K^* strain was estimated to be about 400 RPM (Table S3). We hypothesized that either *I*-specific, or *hsp70* piRNAs, or both could transform *I-TGs* into piRNA-producing loci. However, previously reported evidence is against the role of *hsp70* piRNAs in the stimulation of piRNA production by the cognate *I-TG* transgenes [19,20]. Indeed, none of the ten promoterless transgenes bearing the *I*-sequence without promoter, the *hsp70* polyadenylation sequence instead, and a *mini-white* gene under the control of a *hsp70* promoter demonstrated *I*-silencing activity [20]. In this case, piRNA production from the *hsp70* fragments did not spread into the *I*-fragment. These data suggested an essential if not a key role for the *I*-element-derived piRNAs in the transformation of *I-TG* transgenes into piRNA clusters.

**Figure 3:**
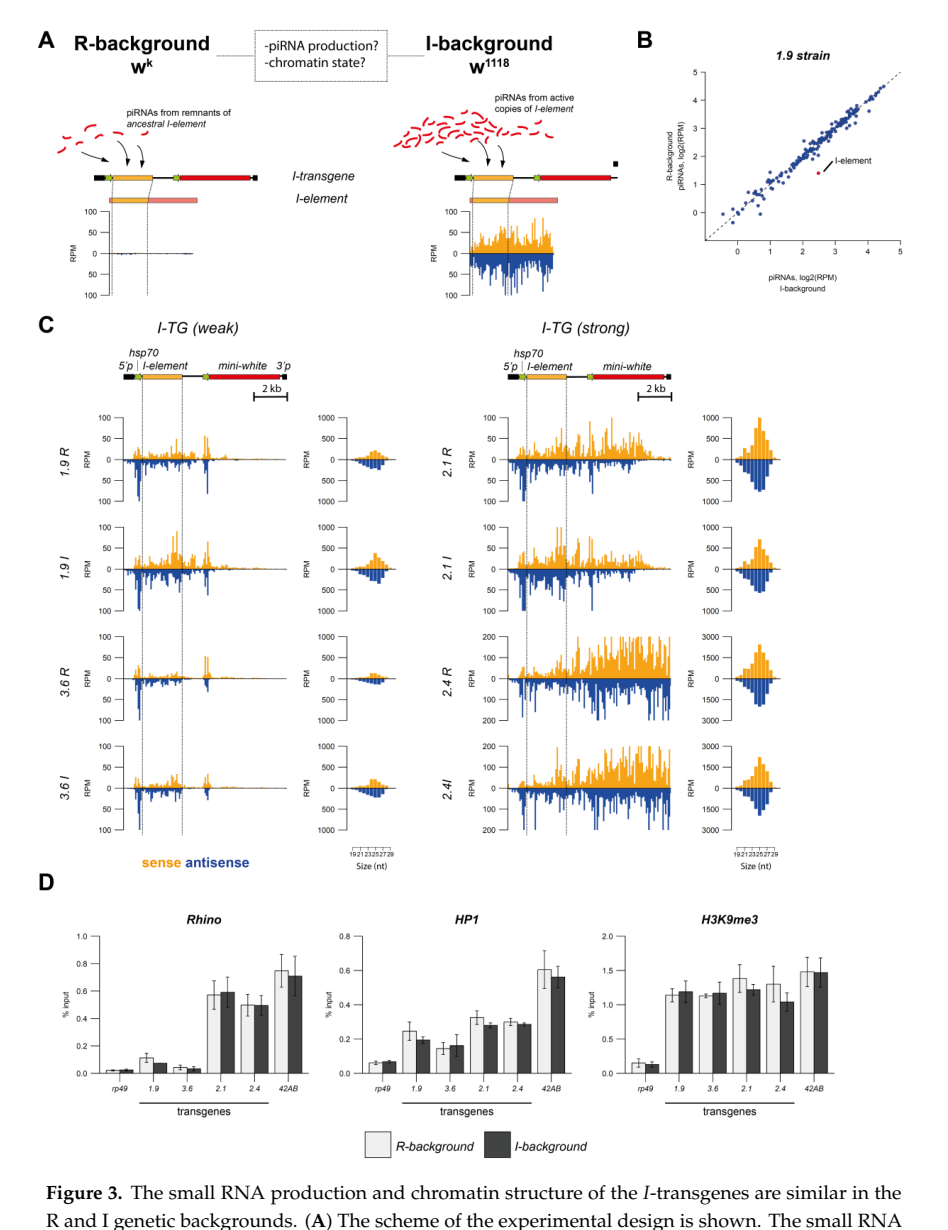
The small RNA production and chromatin structure of the *I*-transgenes are similar in the R and I genetic backgrounds. (**A**) The scheme of the experimental design is shown. The small RNA distribution along the canonical *I*-element in *w^K^* (Reactive (R) strain) and balanced *w^1118^; CyRoi/+;TM3, Sb Ser/+* (Inducer (I) strain, designated as *w^1118^*) is shown below the scheme. (**B**) The scatter plot shows normalized Transposable Element (TE)-specific piRNAs (24–29 nt reads considered, 0-3 mismatches allowed) in ovaries of 1.9^w^R and 1.9^w^I strains. (**C**) Normalized numbers of ovarian small RNAs (19–29 nt) mapped to the indicated transgenic constructs placed in R or I genetic backgrounds (brown—sense; blue—antisense; no mismatches allowed). The scheme of the *I-TG* transgene is shown above. (**D**) HP1, H3K9me3, and Rhi occupancy at the *I-TG* transgenes in R and I genetic backgrounds. The *rp49* and *42AB* regions are used as negative and positive controls, respectively.

We therefore asked if “weak” transgenes could be converted into “strong” dual-strand piRNA clusters if initially targeted by a substantially larger amount of *I*-specific piRNAs. To this end, we performed the paternal transmission of *I-TG* transgenes to the so-called “I-background” by crossing transgene-containing males with females containing functional *I*-elements (Figure 3A). In ovaries of the *I* strain that we used (*w^1118^; CyRoi/+;TM3, Sb Ser/+)*, abundant sense and antisense piRNAs were mapped along the whole *I*-element sequence (Figure 3A); the level of *I*-specific piRNAs that could target the *I-TGs* was ~70 fold higher than in *w^K^* and estimated to be about 2000 RPM (Table S3). Presumably, this pool of maternal piRNAs could convert paternal *I-TG* transgenes into strong piRNA clusters and potentiate piRNA production. To test this idea, we chose “weak” (1.9^w^R and 3.6^w^R) and “strong” (2.1^s^R and 2.4^s^R) transgenes and transferred them from the R-background to the I-background to generate 1.9^w^I, 3.6^w^I, 2.1^s^I, and 2.4^s^I strains (see the Materials and Methods). Ovarian small RNA sequencing of the progeny confirmed the higher level of *I*-specific piRNAs in the I-background, while other TEs were unaffected (Figure 3B, Figure S1). The calculation of small RNAs derived from the non-transgenic fragment of the *I*-element demonstrated a ~20-fold increased amount of these reads at the I-background relative to the R-background (Table S3). This fact confirmed that active *I*-element copies involved in the piRNA production were indeed present in the “I” transgenic strains. The presence of active *I*-element copies in the “I” transgenic strains was also confirmed by tests of the inducer phenotype (see the Materials and Methods), demonstrating on average ~80% levels of embryonic lethality in dysgenic crosses between transgenic “I” males and *w^K^* females (Table S2).

To investigate the differences in transgenic piRNA production between the R and I genetic backgrounds, we mapped small RNA reads to the *I-TG* transgene allowing no mismatches (Figure 3C, Table S3). Surprisingly, we did not detect any significant differences in transgenic piRNA production between the R and I genetic backgrounds, neither for “strong”, nor for “weak” transgenes. A hallmark of transgenic piRNA clusters is the production of piRNAs by the *mini-white* transgenic region [22] caused by the spreading of small RNA production beyond the *I-*fragment. *I-TG* “weak” transgenes produce similarly low amounts of piRNAs from the *mini-white* gene in both the R and I backgrounds. Interestingly, comparison of piRNA mapping to the transgenic *I*-element fragment between the R and I background (allowing 0 and 1–3 mismatches) also did not reveal any substantial differences (Table S3, Table S4). Previously, we reported that piRNAs corresponding to the transgenic *I*-element fragment originated predominantly from the ping-pong piRNA amplification resulting from the interaction between transcripts of transgenes and ancestral *I*-element fragments residing in the *w^K^* genome [19,22]. It could be expected that in the I background, the interaction between the transgenic transcripts and abundant transcripts of the functional *I*-elements would enhance amplification of *I*-specific piRNAs. However, the level of piRNAs mapping to the transgenic *I*-element fragment is not affected by the I background, indicating that this level could have already been saturated in the transgenic R strains. In this case, the piRNA production cannot be optimized by a higher level of endogenous *I*-element transcripts. This suggests that piRNA production is limited either by the capacity of the piRNA processing machinery or by the abundance of the transgenic transcripts.

Next, we studied the chromatin structure of the *I-TG* transgenes in the R and I background. Interestingly, we did not observe any significant differences, neither in H3K9me3 enrichment, nor in Rhi and HP1 recruitment on “weak” and on “strong” transgenes. These results confirmed that the chromatin state of the transgenes was not altered by a ~70-fold higher amount of *I*-specific piRNAs (Figure 3D). Most importantly, the enrichment of Rhi, a key factor that promotes piRNA cluster transcription, did not change between the R and I background. This result was in accordance with the fact that piRNA production from the transgenes also was unaffected.

Overall, these data demonstrated that the ~70-fold enhanced level of piRNAs targeting the transgenic locus did not guarantee a potentiation of the locus into a “strong” piRNA cluster. In addition, these results suggested that piRNAs from the ancestral damaged *I*-elements, which are present in both the R and I background, were likely sufficient to trigger the highest production of piRNAs that was possible from each single transgenic locus. To test this suggestion, we performed the study of paternal and maternal transgene inheritance in the R background.

### 3.3. Transgenic piRNA Cluster Establishment Is Independent of High Levels of Maternally-Transmitted Targeting piRNAs

**Figure 4:**
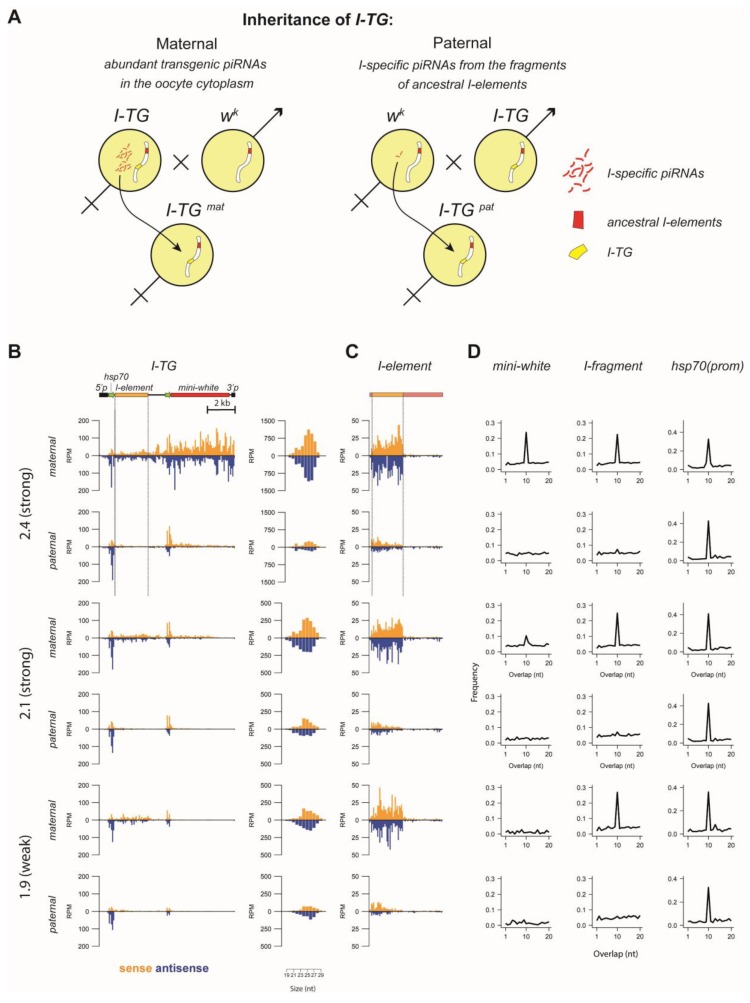
Small RNA production and chromatin structure of maternally- and paternally-inherited *I-TG* transgenes. (**A**) The scheme of the experimental design is shown. (**B**) Normalized numbers (RPM) of ovarian small RNAs (19–29 nt) mapped to the indicated transgenic constructs inherited maternally or paternally (brown—sense; blue—antisense; no mismatches allowed). The scheme of the *I-TG* transgene is shown above. The length distribution of transgenic small RNAs is shown to the right. (**C**) Small RNA distribution along the canonical *I*-element in the R strains after maternal/paternal transgene inheritance. (**D**) Ping-pong signature: the relative frequencies of 5′-overlap length (one to 20 nucleotides) between sense and antisense 24–29-nt piRNAs for the transgenic *I*-fragment, the *mini-white* region, and the *hsp70* promoter are shown.

It is well established that maternally-inherited piRNAs mediate epigenetic changes that can be transmitted through hundreds of generations [18,40]. The R strain *w^K^*, which was used for the *I-TG* transgenesis, is a strong reactive strain, meaning that a very high level of *I*-element transposition was observed when introducing functional *I*-elements by a cross between *w^K^* females and inducer males. In *w^K^* ovaries, only a minimal amount of ancestral *I*-derived piRNAs is produced [19,24,40] (Tables S3 and S4). One could argue that initially, the chromatin structure and piRNA production from the *I*-transgenes in all strains were the same, although later, in the course of the strain-specific history, the transgenes in the 2.1^s^ and 2.4^s^ strains became stronger, which resulted in higher Rhi occupancy and more efficient piRNA production. This could have happened, for example, as a result of the increase in temperature or ageing − epigenetic factors, which are known to affect the transcription of piRNA clusters and the accumulation of piRNAs [41,42,43]. The *I-*transgenes in the 1.9^w^ and 3.6^w^ strains did not demonstrate efficient piRNA production, possibly due to the strain-specific epigenetic history. We therefore decided to simulate the initial conditions for the formation of *I-TG* transgenic piRNA clusters by the paternal inheritance of transgenes. To this end, we crossed 1.9^w^, 2.1^s^, and 2.4^s^ transgenic strains with *w^K^* in order to transmit the transgene either maternally or paternally (Figure 4A). In the case of the paternal inheritance of the transgenes, they would be targeted by negligible amounts of *I*-specific piRNAs inherited with the oocyte cytoplasm of the *w^K^* mothers. In the case of maternal transgene inheritance, a 30-fold higher amount of *I*-specific piRNAs of transgenic origin were deposited from the mother to progeny (Table S3). We sequenced small RNAs and analyzed the chromatin structure of the transgenes in the ovaries of the F1 progeny.

First, small RNA-seq revealed substantial differences between transgenic small RNA profiles for the paternally- (1.9^w^pat, 2.1^s^pat, and 2.4^s^pat) and maternally- (1.9^w^mat, 2.1^s^mat, and 2.4^s^mat) inherited transgenes (Figure 4B). Indeed, there were much smaller RNAs mapping the transgenes after paternal introduction of the transgene than after maternal introduction (Figure 4B). In all cases, we observed small RNAs mapping to the *I*-fragment. These piRNAs were clearly transgene-specific since only the fragment of the *I*-element, which is present in the transgene, generated substantial piRNA levels compared to the other *I*-element regions (Figure 4C).

After maternal inheritance, transgenic small RNA profiles resembled those of the corresponding initial R transgenic strains (Figure 3C, Figure 4B,C). The *mini-white* region downstream of the *I*-fragment produced abundant piRNAs in 2.4^s^mat and 2.1^s^mat (Figure 4B). piRNAs generated by the ping-pong mechanism were characterized by the so-called ping-pong signature, an over-representation of 10 nucleotide 5’-overlaps between sense and antisense piRNAs [1,23]. In the case of maternal transmission, this ping-pong signature was clearly present for the *I*-fragment for each transgene, as well as for the *mini-white* region for 2.4^s^mat and 2.1^s^mat (Figure 4D). For the 1.9^w^mat transgene, *mini-white* produced only very few piRNAs and showed no ping-pong signature. In the case of paternal transmission, there was no significant ping-pong signature, neither for the *I*-fragment, nor for the *mini-white* region. In contrast to 1.9^w^pat and 2.1^s^pat, the 2.4^s^pat transgene was able to produce piRNAs from the *mini-white* region of the transgene while being targeted by the same pool of maternal *w^K^* piRNAs (Figure 4B, Table S3). *Mini-white* piRNAs lacked the ping-pong signature, but showed a strong 5′ terminal uridine (1U) bias (62%), a signature of primary piRNAs [1,44]. Thus, the entire 2.4^s^pat transgene behaved like a piRNA cluster even without substantial targeting by piRNAs inherited from the mother.

**Figure 5:**
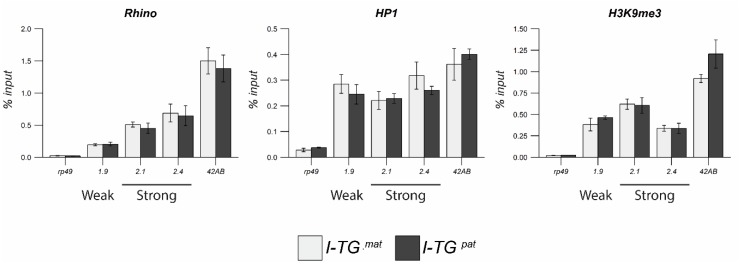
Chromatin structure of maternally- and paternally-inherited *I-TG* transgenes. HP1, H3K9me3, and Rhi occupancy at maternally- and paternally-inherited *I-TG* transgenes. The *rp49* and *42AB* regions are used as negative and positive controls, respectively.

Next, we performed ChIP-qPCR to compare the chromatin of paternally- and maternally-inherited transgenes using primers corresponding to the 5′P region of the transgenes (Figure 5). Surprisingly, we did not observe any significant differences, suggesting that the heterochromatin components (HP1 and H3K9me3) and piRNA cluster factor Rhi were deposited at paternally-inherited transgenes at the same levels as at maternally-transmitted transgenes. Presumably, a low level of maternal *I*-specific piRNAs transmitted from R females was sufficient to ensure H3K9me3 markers and HP1 binding for all transgenes. It is noteworthy that a ~3-fold higher level of Rhi was deposited at paternally-inherited 2.1^s^ and 2.4^s^ transgenes than at 1.9^w^. These results argued that the establishment of the piRNA-cluster heterochromatin state and production of primary piRNAs were regulated by the “all-or-nothing” rule, since the few maternally inherited piRNAs mapping to the *I*-element (<30 reads) derived from *w^K^* were sufficient for this.

**Figure 6:**
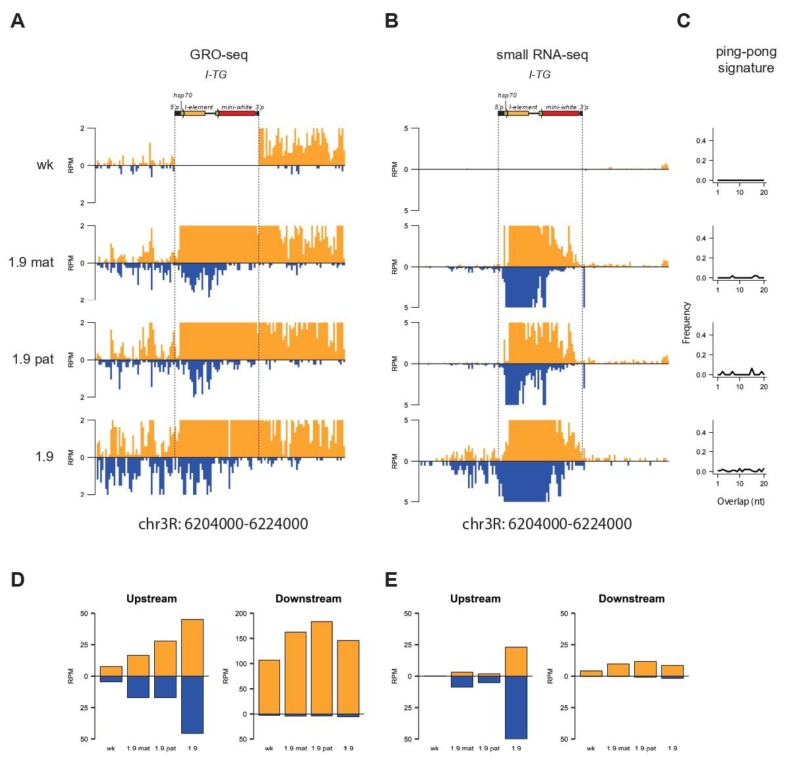
Analysis of Global Run-On sequencing (GRO-seq) performed on ovaries of R strains after maternal or paternal inheritance of the 1.9 *I-TG* transgene. (**A**) Normalized GRO-seq reads mapping to the 1.9^w^ transgene insertion region (± 10 kb) in 1.9^w^mat, 1.9^w^pat, 1.9^w^, and 3.1 (designated as *w^K^*) strains (brown—sense; blue—antisense, no mismatches allowed). The 3.1 strain contained the *I-TG* transgene in subtelomeric region and was used here as a control for the 1.9^w^ transgenic strain. Genome coordinates are given according to the Drosophila R5 release. (**B**) Small RNA-seq reads (19–29 nt, brown—sense; blue—antisense) mapping at the same region and in the same strains as in (**A**). (**C**) Ping-pong signature: Relative frequencies of 5′-overlap length (one to 20 nucleotides) between sense and antisense 24–29-nt small RNAs mapping to the upstream region (10 kb) of the 1.9^w^ transgene insertion sites in the same strains as in (**A**). (**D**) Normalized counts of GRO-seq reads mapping to the upstream and downstream regions of the 1.9^w^ transgene insertion site (± 10 kb). (**E**) Counts of small RNA-seq reads (19–29 nt) mapping to the upstream and downstream regions of the 1.9^w^ transgene insertion site (± 10 kb).

We previously showed that the 1.9^w^ transgene insertion caused the transformation of the upstream flanking region into a dual-strand mixed si/piRNA cluster accompanied by an increased transcription of both genomic strands [22]. Here, we aimed to compare the transcription profile and distribution of unique small RNAs at this genomic region after maternal or paternal transmission of the 1.9^w^ transgene. For this, Global Run-On sequencing (GRO-seq) allowing the measurement of nascent RNA synthesis was performed on ovaries from F1 progeny bearing 1.9^w^mat or 1.9^w^pat. As an “empty” site, we used the same genomic region in the 3.1 strain, which did not contain the transgene in this site [22]. In both cases, we observed a similarly modest transcriptional upregulation from both genomic strands in the upstream transgene flanking region that was silent in the absence of the transgene. The initiation of convergent transcription in the transgene upstream region was accompanied by the production of small RNAs lacking the ping-pong signature, but demonstrated 5′ uridine (1U) bias (70%) typical for primary piRNAs (Figure 6). Thus, it seemed that the 1.9^w^ transgene promoted similar transcriptional changes in the flanking region independently of the levels of maternally-transmitted piRNAs targeting the transgene. Taken together, our data suggested that the primary attributes of piRNA clusters such as chromatin structure, primary piRNA production, and initiation of convergent transcription did not depend on the starting amount of piRNAs targeting the transgene. Still, acting on the established transgenic piRNA clusters, abundant maternal piRNAs stimulated piRNA production from the transgenes through ping-pong amplification.

Thus, a minimal level of maternal transgene-specific piRNAs was sufficient to mediate the paramutation of the transgenes and establish piRNA clusters. The manifestation of paramutation most likely depended on the genomic context of the *I-TG* transgene insertion sites.

### 3.4. Comparison of the Transcription Status of I-Transgene Localization Sites

**Figure 7:**
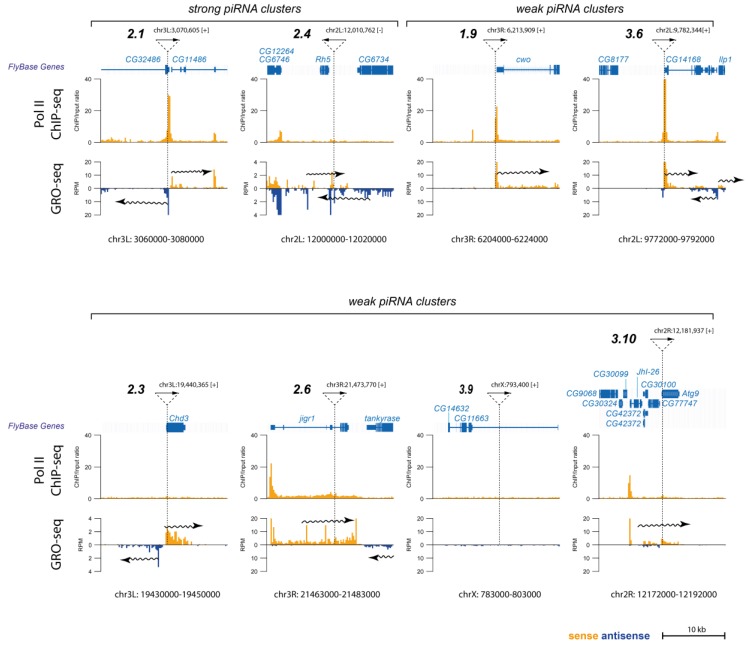
Transcriptional status of *I-TG* transgene insertion loci before insertion of the transgenes. PolII ChIP-seq data are shown as a ChIP/input ratio. GRO-seq reads mapping to the genomic loci of *I-TG* transgene insertions are shown in RPM. The pictures were generated using the UCSC Genome Browser. Dotted lines indicate the insertion sites of the *I-TG*s. Plain arrows indicate the genomic orientation of transgenes; wavy arrows show the direction of transcription in a locus.

Our data suggested that the insertion sites of “weak” transgenic piRNA clusters were likely to be resistant to Rhi binding and piRNA cluster formation. Despite the identical sequence composition, transgenes displayed different piRNA-producing patterns, suggesting a crucial role for the genomic context of the transgene insertion site. Presumably, some genomic regions were more susceptible to piRNA-directed paramutations (2.1^s^ and 2.4^s^ insertion sites), whereas the others were less (1.9^w^ and 3.6^w^ insertion sites). To seek the nature of the differences between transgene insertion sites, we analyzed published ovarian PolII ChIPseq [3] and GRO-seq [22] data. Other previously described *I-TG* transgenes (2.3, 2.6, 3.9, 3.10) [19] were added to the analysis. All of them could be attributed to “weak” piRNA clusters because piRNA production did not spread beyond the *I*-element fragment [19]. The transgenic system described here was based on P-element vectors, the insertions of which showed a strong correlation with replication origins frequently overlapping with promoters [45]. Indeed, insertion sites of several *I-TG* transgenes including 1.9^w^, 3.6^w^, and 2.1^s^ piRNA clusters localized within PolII peaks (Figure 7). At the same time, insertions of 2.4^s^, 2.3^w^, and 3.9^w^ transgenes did not coincide with PolII peaks, indicating no relationship between PolII enrichment and *I-TG* transgene behavior. Next, we performed the analysis of our previously published GRO-seq data obtained on ovaries with the *w^K^* genetic background, but lacking euchromatic transgene insertions [22]. GRO-seq provides more information about the transcriptional status of genomic loci since it allows assaying the levels of strand-specific nascent RNA synthesis. Specifically, we focused on the transcription at the transgene insertion sites (“empty” sites in non-transgenic *w^K^*). For “weak” transgenes (1.9^w^, 3.6^w^, 2.3^w^, 2.6^w^, 3.9^w^, 3.10^w^), we observed that the transgenes and the genomic loci were transcribed in the same direction. In the case of 2.1^s^ and 2.4^s^ transgenes, GRO-seq revealed transcription of genomic loci in the direction opposed to the transgene transcription (Figure 7). The 2.1^s^ transgene was transcribed in the opposite direction to the *CG32486* gene in which it was inserted. The region of the 2.4^s^ insertion was transcribed in both directions, which was likely favorable for the initiation of the convergent transcription of the transgene.

We speculated that convergent transcription may facilitate establishment of dual-strand piRNA clusters characterized by the transcription of both genomic strands. However, the low number of “strong” transgenic strains did not allow an unambiguous conclusion.

## 4. Discussion

**Figure 8:**
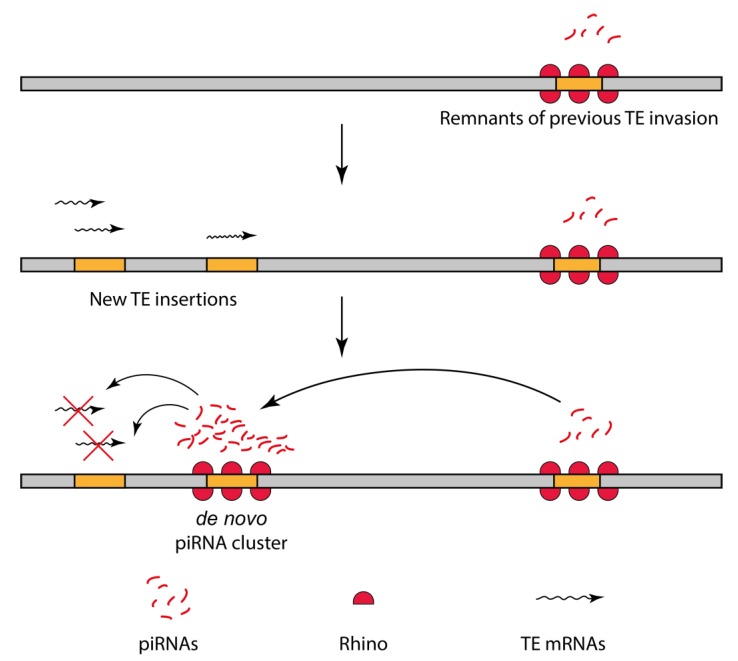
A model of *de novo* piRNA cluster formation. A low amount of endogenous piRNAs can cause *de novo* formation of a robust piRNA cluster at the cognate sequences, thus boosting the RNAi response against genome invaders.

An important question concerning piRNA-producing loci is how they evolved to become efficient piRNA clusters. The transgenic model of piRNA clusters is a powerful tool to study the genomic and epigenetic prerequisites of *de novo* piRNA cluster formation. Here, we addressed an essential question regarding the quantitative level of piRNAs required for triggering piRNA production from the cognate locus. Our transgenic system simulated the natural situation of genome invasion by alien transposons. The current model of piRNA cluster establishment implies binding of the Piwi-piRNA complex to complementary RNA cotranscriptionally. We showed that targeting the transgenic locus by an enhanced level of piRNAs did not transform “weak” piRNA clusters into “strong” ones and did not enhance piRNA production from “strong” piRNA clusters. A minimal level of *I*-element piRNAs, which were present in R strains, was enough to turn on piRNA-mediated heterochromatinization and piRNA cluster establishment. It seemed that the effector function of piRNAs works according to the “all-or-nothing” rule. The prompt response of the piRNA system to the expansion of piRNA targets appears to be beneficial in a population because it ensures transposon suppression at early stages of their propagation and, hence, provides evolutionary advantages. According to our model, the *de novo* formation of piRNA clusters at newly inserted active transposon copies, launched by a scarce level of cognate piRNAs, led to enhanced piRNA production and effective silencing under the conditions of the transposon reintroduction into the genome (Figure 8).

Maternal piRNAs play an essential role in the epigenetic silencing of their targets over generations. The maternally-transmitted piRNA pool deposited in the oocyte launches piRNA processing in the progeny, leading to transposon suppression in *Drosophila* [40]. Maternal inheritance of transgenic piRNAs was reported to be crucial for the paramutation of a homologous initially inactive transgene [18,46]. The latter transgenic model is based on the T1 transgene cluster bearing tandem repeats of *P-lac-w* transgenes located in the heterochromatin of a rearranged chromosome 2R [47]. The T1 locus demonstrated spontaneous conversion into a piRNA cluster able to silence homologous transgenes [18]. Maternally-transmitted transgenic piRNAs were necessary to maintain piRNA production and heterochromatin state at the T1 locus, while paternal transmission of T1 resulted in decreased levels of the H3K9me3 and Rhi at the transgene [18,48]. In contrast, the chromatin of *I-TG* transgenes was not sensitive to the direction of crosses, probably because a low level of endogenous ancestral *I*-element piRNAs transmitted with the oocyte (<30 RPM) was sufficient to activate heterochromatinization and Rhi deposition at paternal *I-TG*s. Alternatively, one may suggest that the inheritance of Rhi-, HP1-, and H3K9me3-occupancy, i.e., maintenance of the specific chromatin structure of the transgenes through generations, is independent of maternally-transmitted piRNA and is governed by a yet-unknown mechanism of genomic imprinting. The former explanation seems more believable and implies that abundant transgenic piRNAs and scarce ancestral *I*-related piRNAs inherited from the *w^K^* female have equal capacity to induce chromatin changes and piRNA production by *I-TG* transgenes in the progeny.

Thus, endogenous *I*-related piRNAs in R strains were able to induce robust transcriptional silencing mediated by HP1 and H3K9me3 assembly at *I-TG*s in all studied transgenic strains. However, only two of ten transgenes were converted into “strong” piRNA clusters judging by the spreading of piRNA production beyond the *I*-element fragment into other transgenic regions [19]. “Weak” transgenic clusters targeted by abundant homologous piRNAs were nevertheless not enhanced in terms of piRNA production and Rhi binding. These data stressed the importance of the genomic context in the piRNA cluster establishment. Despite the fact that many piRNA clusters in flies reside in the pericentromeric heterochromatin [1], euchromatic piRNA clusters associated with transposon insertions were also revealed in flies, mosquitoes, and mammals [16,17,49]. Interestingly, in *Drosophila*, only 20% of active euchromatic TEs convert into piRNA clusters [16], suggesting that many euchromatic regions are resistant to the chromatin and transcriptional changes accompanied by piRNA cluster establishment. Similar statistics (two “strong” piRNA clusters of ten analyzed) were observed for the *I-TG* transgenes [19]. Previously, we reported that insertion sites of all studied *I-TG*s were devoid of HP1, Rhi, and H3K9me3, rejecting the possibility that some transgenes were introduced in the appropriate piRNA cluster-specific chromatin environment [22]. However, even the artificial tethering of Rhi to a reporter gene did not trigger piRNA production from the target locus [50], suggesting that *de novo* piRNA cluster formation requires more than just Rhi binding.

*I-TG* piRNA clusters are related to the dual-strand clusters, which are transcribed from both genomic strands and therefore produce sense and antisense piRNAs. Establishment of *I-TG* clusters accompanied by *de novo* activation of antisense transcription results in non-canonical convergent transcription [22]. Most likely, this transcription mode leads to local conformation disorder and interferes with gene expression. We suggest that the transcription status of the insertion site is the most important factor affecting the *I-TG* transgene fate. Indeed, analysis of ovarian GRO-seq data revealed that “strong” transgenes are transcribed in the opposite direction relative to the transcription in the insertion locus. Such a transcription pattern could facilitate convergent transcription, leading to the formation of very efficient transgenic piRNA clusters.

## 5. Conclusions

piRNAs play a key role in the control of TE activity mediating their transcriptional, cotranscriptional and posttranscriptional silencing. piRNA-mediated *de novo* piRNA cluster formation is another level of defense which results in the appearance of novel piRNA source loci in the genome. In our study we discovered that the quantitative threshold of maternal piRNAs required for triggering piRNA production from the cognate locus is very low and estimated as <30 RPM. The capacity of a small pool of piRNAs to induce *de novo* formation of a robust piRNA clutser at cognate sequences is equal to the capacity of a much larger piRNA supply. The formation of *de novo* piRNA clusters appears to boost the piRNA response against genome invaders. The essential question in this field of research is what is the role of genomic context to form an active piRNA cluster. Our data hint that the transcription status of the targeted locus could be an important factor affecting the establishment of the piRNA cluster in the locus. Further whole-genome analysis is needed to address this essential question. 

## Supplementary Materials

The following are available online at https://www.mdpi.com/2073-4409/9/4/922/s1: Figure S1: (Related to Figure 3) The levels of *I*-specific piRNAs in the transgenic strains. The scatter plots show normalized TE-specific piRNAs (24–29 nt reads were considered, 0 or 0–3 mismatches allowed) in the ovaries of the indicated transgenic strains at the R and I background. Table S1: Genomic coordinates and orientation of *I-TG* transgene insertions. Table S2: A test of the inducer phenotype of transgenic strains at the I background. Table S3: Transgenic small RNA content at different genetic backgrounds (0 mismatches). Table S4: Transgenic small RNA content at different genetic backgrounds (1-3 mismatches). Table S5: Primers used in the study (5’ to 3’).
