# Supplementary material for: Epigenetic Requirements for Triggering Heterochromatinization and Piwi-Interacting RNA Production from Transgenes in the Drosophila Germline
Source: Cells. 2020 Apr 10;9(4):922. doi: 10.3390/cells9040922 (PMC7226800; doi:10.3390/cells9040922)
Supplement: Supplementary file 1 [file cells-09-00922-s001.pdf]

Supplementary Figure S1 and Tables S1-S5

Epigenetic requirements for triggering heterochromatinization and Piwi-interacting RNA production from transgenes in the *Drosophila* germline

Running title: establishment of transgenic piRNA clusters

Pavel A. Komarov[1*], Olesya Sokolova[1], Natalia Akulenko[1], Emilie Brasset[2], Silke Jensen[2,3] and Alla Kalmykova[1,3]

[1] Institute of Molecular Genetics, Russian Academy of Sciences, Moscow 123182, Russia

[2] GReD, Université Clermont Auvergne, CNRS, INSERM, Faculté de Médecine, TSA 50400, 63001 Clermont-Ferrand Cedex 1, France

[3] **Corresponding authors:** Alla Kalmykova, Institute of Molecular Genetics of Russian Academy of Sciences, Kurchatov sq. 2, 123182 Moscow, Russia; phone: 7(499) 1960019; fax: 7(499)1960221; e-mail: allakalm@img.ras.ru

Silke Jensen, GReD, Université Clermont Auvergne, CNRS, INSERM, Faculté de Médecine, TSA 50400, 63001 Clermont-Ferrand Cedex 1, France. Tel: 0033-4-7317-8184 Fax: 0033-4-7327-6132 e-mail: silke.jensen@uca.fr

* Present address: Friedrich Miescher Institute for Biomedical Research, Maulbeerstrasse 66, 4058 Basel, Switzerland

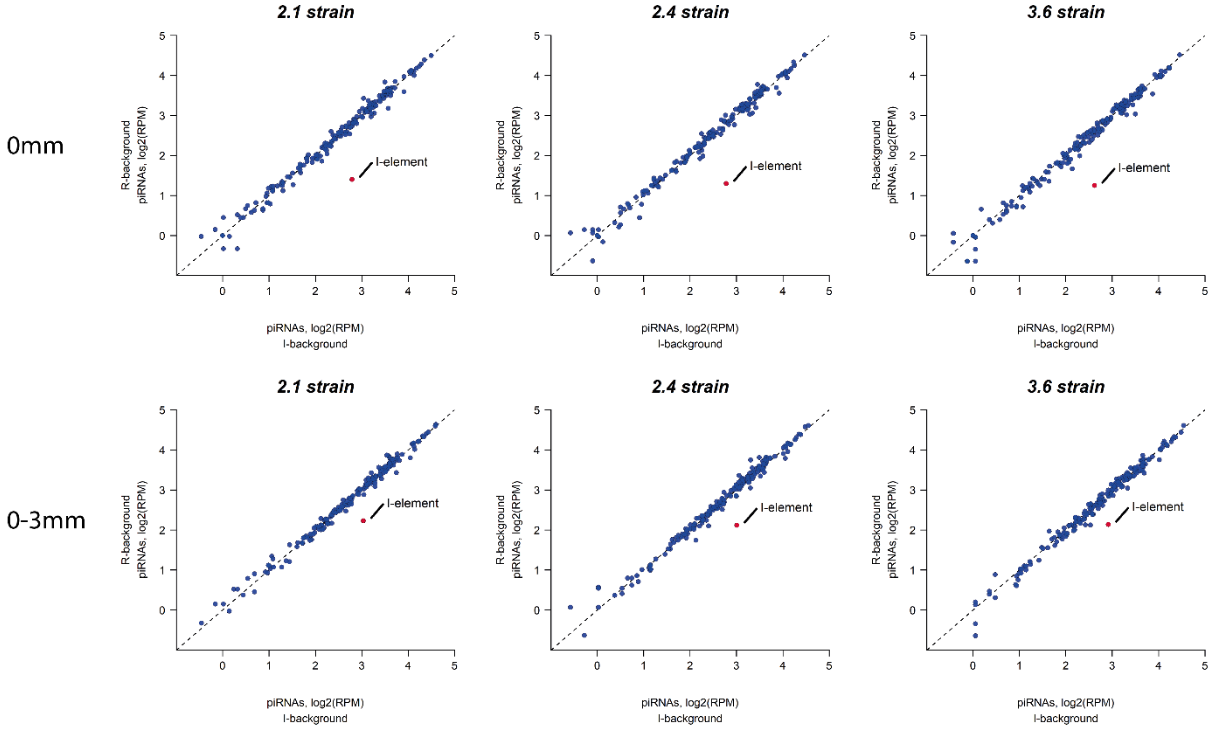

Figure S1 (related to Figure 3). The levels of I-specific piRNAs in the transgenic strains. Scatter plots show normalized TE-specific piRNAs (24-29 nt reads were considered, 0 or 0-3 mismatches allowed) in ovaries of indicated transgenic strains at R and I background.

Tables S1-S5

Table S1. Genomic coordinates and orientation of I-TG transgene insertions

Strain	Insertion site
1.9	chr3R:6213909 [+]
2.1	chr3L:3070605 [+]
2.4	chr2L:12010762 [-]
3.6	chr2L:9782344 [+]

Table S2. A test of the inducer phenotype of transgenic strains at I background

femalexmale	% of dead embryos (N>100)
wKx1.9I	89
wKx2.4I	73
wKx2.1I	60
wKx3.6I	72
wKxw1118	94

Table S3. Transgenic small RNA content at different genetic backgrounds (0 mismatches). Normalized numbers of sense and antisense small RNAs (19-29 nt) mapped to the indicated regions with 0 mismatches allowed. "Transgenic I-fragment", "*mini-white*" and "*hsp70*" are fragments that are present in the transgenes, "non-transgenic I fragment" is the *I*-element region that is absent from the transgenes.

Strain	*orientation*	*mini-white*	*hsp70*	*transgenic I-fragment*	*non-transgenic I-fragment*
w^k	S	0	129	11	6
	AS	0	270	17	17
w^{1118}	S	6	21	832	1261
	AS	3	48	1084	1413
1.9R	S	78	116	411	16
	AS	23	411	474	23
1.9I	S	115	113	728	143
	AS	28	465	763	263
3.6R	S	57	107	204	12
	AS	7	282	351	21
3.6I	S	49	83	325	191
	AS	3	263	435	354
2.1R	S	899	128	733	13
	AS	295	579	765	27
2.1I	S	565	160	1044	244
	AS	273	684	1212	614
2.4R	S	4320	204	1072	12
	AS	2815	999	1404	22
2.4I	S	3636	140	1228	209
	AS	2554	747	1662	604
1.9^{mat}	S	37	75	375	
	AS	4	316	311	
1.9^{pat}	S	40	79	118	
	AS	4	319	45	
2.1^{mat}	S	234	131	408	
	AS	71	419	393	
2.1^{pat}	S	74	130	110	
	AS	7	339	87	
2.4^{mat}	S	1835	121	481	
	AS	1373	667	594	
2.4^{pat}	S	324	119	137	
	AS	100	392	106	

Table S4. Transgenic small RNA content at different genetic backgrounds (1-3 mismatches). Normalized numbers of sense and antisense small RNAs (19-29 nt) mapped to the indicated regions with 1-3 mismatches allowed. Column designations are as in Table S3.

Strain	*orientation*	*mini-white*	*hsp70*	*transgenic I-fragment*	*non-transgenic I-fragment*
w^k	S	0	31	15	11
	AS	0	58	31	33
w1118; CyRoi/+; TM3, Sb Ser/+	S	0	5	201	221
	AS	0	12	289	369
1.9R	S	7	20	109	26
	AS	2	72	154	36
1.9I	S	9	30	137	72
	AS	2	84	187	121
3.6R	S	4	16	101	21
	AS	1	57	143	37
3.6I	S	2	22	127	85
	AS	0	66	134	124
2.1R	S	56	24	124	27
	AS	11	111	195	45
2.1I	S	30	27	146	81
	AS	17	88	256	179
2.4R	S	246	41	151	15
	AS	175	127	272	36
2.4I	S	217	31	163	76
	AS	164	107	290	156
1.9^{mat}	S	4	21	161	
	AS	1	76	192	
1.9^{pat}	S	5	26	48	
	AS	1	86	39	
2.1^{mat}	S	8	21	106	
	AS	3	63	176	
2.1^{pat}	S	3	18	33	
	AS	0	56	52	
2.4^{mat}	S	150	26	112	
	AS	105	118	173	
2.4^{pat}	S	25	25	33	
	AS	8	80	44	

Table S5. Primers used in the study (5' to 3')

name/target	sequence
rp49	GACGCTTCAAGGGACAGTATCTG
	AAACGCGGTTCTGCATGAG
42AB	CGTCCCAGCCTACCTAGTCA
	ACTTCCCGGTGAAGACTCCT
HeT-A 3'UTR	TGAAAGAGATACCCACCAAATGACAGC
	CCAGAAGGACGGAAGCACCAAGT
5'P	AGAGGAAAGGTTGTGTGCGGAC
	ACCCAAGGCTCTGCTCCCACA
Mini-white	AGTTGCACTTTGTCAGCGGTTTCG
	CCCCTGCTTACCCACCCAAAA
2.4_ins_up	GGTGGGTAGATCGAGTCCGGATACC
	GCAAGAAGAAGCCCCACTTTGCG
